# Health effects of microplastics and nanoplastics: review of published case reports

**DOI:** 10.5620/eaht.2024020

**Published:** 2024-06-21

**Authors:** Oche Joseph Otorkpa, Chinenye Oche Otorkpa

**Affiliations:** 1 Department of Public Health, School of Public Health, Texila American University, Georgetown, Guyana; 2 Department of Physiology, College of Health Sciences, Federal University, Lokoja, Nigeria

**Keywords:** microplastics, nanoplastics, toxicity, exposure, health effects

## Abstract

Microplastics and nanoplastics (MNPs) represent a pervasive environmental pollutant, raising significant concerns about potential health effects on humans. These tiny plastic particles have been detected across various environmental matrices, including air, water, soil, and food sources. While the adverse impacts of MNPs on wildlife and ecosystems are well-documented, understanding their effects on human health is still in its infancy. This study aims to comprehensively review existing case reports documenting adverse health outcomes associated with MNPs exposure. Through an extensive literature search, relevant articles were identified and analyzed. MNPs exposure primarily occurs through ingestion and inhalation routes. Health effects on the digestive system include oxidative stress, inflammation, dysbiosis, and metabolic disorders, with cases linking MNPs exposure to gastrointestinal injury and liver dysfunction. Respiratory system impacts include asthma exacerbation and hypersensitivity pneumonitis, particularly in industries involving plastic production. MNPs exposure has also been associated with nervous system conditions, reproductive toxicity, skeletal system interference, excretory system disruption, and cardiovascular morbidity and mortality. Despite limited case reports, the widespread presence of MNPs warrants further investigation into their potential health risks. This study underscores the urgency of understanding and mitigating the adverse health effects posed by MNPs exposure. Further research is imperative in order to comprehensively assess and address the dangers associated with MNPs contamination in the environment.

## Introduction

Microplastics are small plastic particles, usually below 5 millimeters in size, which originate from the fragmentation of larger plastic particles or are intentionally manufactured at a microscopic scale [1]. These particles encompass a wide range of materials, including polyethylene, polypropylene, polystyrene, and others, and can take various forms such as fragments, fibers, pellets, or microbeads [2]. Nanoplastics on the other hand are extremely small plastic particles, typically ranging from 1 nanometer to 100 nanometers in size [3]. These minuscule particles can be either be intentionally manufactured at the nanoscale or unintentionally produced from the degradation of larger plastic items or microplastics. Nanoplastics can be composed of various polymers, including but not limited to polyethylene, polypropylene, polystyrene, and polyvinyl chloride [4].

The pervasive presence of MNPs in the environment has raised significant concerns regarding their potential effects on the health of humans. These minuscule plastic particles, measuring less than 5 millimeters in size, have been detected in various environmental matrices, including air, water, soil, common food sources and even bottled water [5]. While the adverse impacts of MNPs on wildlife and ecosystems are well-documented, the understanding of the health effects of microplastics and nanoplastics on humans is still in its infancy. MNPs have been also been reported in various biological samples, including feces, sputum, saliva, blood, and even the placenta. These tiny plastic particles have been associated with the development or exacerbation of a wide range of health conditions, including cancer, intestinal issues, lung problems, heart disease, infectious diseases, and inflammatory conditions [6].

Given the increasing prevalence of plastic pollution and its potential consequences for human health, there is a pressing need to comprehensively review and analyze existing but scarce case reports documenting adverse health outcomes associated with exposure to MNPs. By synthesizing available evidence from published case reports, this study aims to elucidate the potential health risks posed by these particulate pollutants and identify gaps in current knowledge that warrant further research.

## Methodology

An internet search was conducted using the PubMed and Google Scholar data bases. Medical Subject Headings (MeSH) terms related to microplastics, nanoplastics, health effects, case reports were used to retrieve relevant articles from PubMed, while a free text search of the topic was used for Google scholar. The Boolean search strategy combining keyword and MeSH terms for the main variables was used to improve the yield of the study. The search terms used were as follows:

[‘Case Histories’ OR ‘Case Reports’ OR ‘Case Studies’ OR ‘Case Study’] for case reports; [‘Health effects’ OR ‘Health impacts’ OR ‘Health consequences’] for health effects; [‘Microplastics’ OR ‘Mesoplastics’ OR ‘Plastic Microparticles’ OR ‘Plastic Nanoparticles’] for Microplastics and Nanoplastics. The search terms were entered into the databases with an ‘AND’ term between each of them. The search encompassed articles published from January 2004 to April 2024. The studies with relevant topics and their abstracts written in English language identified by the above search methods were collated together from the different data bases and uploaded into the Rayaan website.

After upload of the identified relevant topics and their abstracts into the Raayan website, duplicates were easily identified based on a minimum of 96% similarity score and deleted after cross-check and confirmation. Following the removal of duplicate articles, all full-length papers and English-language papers were retained for analysis. The titles and abstracts of retrieved articles were screened to identify potentially relevant studies. A total of 85 articles were gathered. Included studies were those reporting case reports or case series describing health effects attributed to exposure to microplastics or nanoplastics and their components while excluded studies were those not reporting health outcomes or studies lacking detailed information on exposure and health effects.

The methodological quality of included case reports was assessed using established criteria adapted from the CARE (CAse REport) guidelines. This assessment considered aspects such as clarity of reporting, appropriateness of each study, documentation of clinical outcomes, and any potential biases. This review adhered to ethical standards regarding the use of published data, ensuring confidentiality and anonymity of patients included in case reports. No primary data collection involving human subjects was conducted as part of this study.

### Health Effects on the Digestive System

One of the prime targets of MNPs toxicity is the digestive system primarily through the overproduction of reactive oxygen species which triggers injury to the digestive system leading to detrimental health effects, such as oxidative stress, inflammation, apoptosis, dysbiosis and several metabolic disorders that often results in increased rates of digestive morbidity and mortality [7]. A major source of MNPs ingestion in children occurs via plastic baby bottles according to a new study. The authors reported that plastic baby bottles with warm water released up to 16 million microplastics per liter. The researchers estimated that children who are fed using plastic baby bottles could potentially be exposed to about 14,600 to 4,550,000 particles per capita per day, which corresponds to a dietary intake of MNPs ranging from approximately 2.3 to 707.5 micrograms per day, based on an average particle mass of 50 nanograms, although the exact level of exposure may vary depending on the region [8].

A prospective case series reported inadvertent ingestion from different sources remains as a major source of MNPs introduction into the adult human body [9]. A study that investigated acute toxicological effects of polystyrene and polyvinyl chloride MNPs reported that during active inflammatory processes, exposure to polyvinyl chloride particles were found to cause a loss of epithelial cells and intestinal injury [10]. A notable case report involved 42-year-old South Asian who presented following self-ingestion of Polyvinyl chloride (PVC)-based solvent cement (S-lon® ). However, a few hours later, the patient experienced central nervous system depression and stridor, leading to the need for intubation. Examination showed inflammation and swelling in the upper airway. The patient was sedated, ventilated, and given intravenous dexamethasone. Attempts to remove the nasogastric tube failed due to the formation of a solid clump, likely formed by a reaction with the substance ingested. The patient had to be reintubated and sedated due to extreme agitation. After the clump was removed, the patient fully recovered and was transferred to the ward after five days. Ingesting PVC)-based solvent cement could result in gut absorption with central nervous system depression, coma, and even death [11]. MNPs from the PVC resin itself or from additives present can also persist for a long time gastrointestinal tract following ingestion and prolonged absorption of vinyl chloride by the intestinal lining can induce hepatotoxicity and hepatic cancers, including angiosarcoma [12].

MNPs ingestion has also been associated with gut microbiome disruption and can trigger local inflammatory and immune responses. In addition, MNPs can serve as carriers of contaminants and enhance the effects of toxic substances, acting as "Trojan Horses." [13].

Another major effect of MNPs on the digestive system is its impact liver function, studies have shown that MNPs can negatively impact liver function via oxidative stress and changes in cell function since the liver is the biggest digestive organ and a main gateway for MNPs to enter the body. MNPs have also been linked with clinical diseases such as metabolic dysfunction-associated fatty liver disease, steatohepatitis, liver fibrosis, and cirrhosis [14].

### Health Effects on the Respiratory System

Inhalation represents another major route of exposure, particularly for airborne microplastics and nanoplastics. These particles can become airborne through processes such as fragmentation of larger plastic items, abrasion of plastic surfaces, or the release of microfibers from textiles. Once airborne, these particles can be inhaled by humans and animals, potentially reaching the respiratory system. The surface area of the lungs' alveoli is around 150 square meters, and the tissue barrier there is less than 1 micron thick. This thin barrier allows nanoparticles (NPs) to pass through into the capillaries, making it easier for them to spread throughout the body [15].

A case of asthma was reported after exposure to a thermoplastic used in 3D printing. The patient, was a businessman who was 28 years and had childhood asthma which resolved by age eight, but began experiencing cough and difficulty in breathing after he started using a 10 fused deposition modeling 3D printers equipped with acrylonitrilebutadiene-styrene filaments within a small work area. In response to this, significant changes was made to his work environment after three months. Some of the changes included switching to polylactic acid filaments and organic cartridge, reducing the number of printers and installing a high efficiency particulate air filter. Even though he slowly improved, he still needed occasional treatment with a salbutamol containing inhaler [16].

3D printing processes and waste poses an environmental health concern as it releases microplastics into the air during the process of melting plastic filament to create objects. These microplastics, can originate from both the filament material, the wear and tear of printer nozzles, as well as the waste associated with the printing process. [17].

In 2020, another case report described a 66-year-old patient who developed hypersensitivity pneumonitis after working in the Polyethylene terephthalate (PET) production industry for many years. The patient had also been exposed to asbestos before. In 2012, the patient was diagnosed with asbestosis and pleural plaques, and by 2017, experienced a minor progression of pulmonary fibrosis alongside inspiratory crackles and decreased lung function. Having worked in a chemical company producing PET from 1992 to 2013, the patient was diagnosed with occupational hypersensitivity pneumonitis (OHP), likely caused by exposure to terephthalic acid and dimethyl terephthalate. [18]. While direct poisoning from terephthalic acid and dimethyl terephthalate are the major culprits in this case , studies have also shown that the breakdown of PET products can result in the release of microplastics which can also exacerbate the condition and lead to other hypersensitivity pneumonitides associated with plastic manufacturing [19].

Styrene a chemical compound commonly used in the production of plastics, including polystyrene which is commonly found in packaging materials, insulation, disposable cups, and food containers has also been implicated in many MNPs related conditions. In 2017, a comprehensive review examined 55 articles including 2 unpublished case reports to investigate the relationship between exposure to styrene and the development of non-malignant lung disorders. The analysis revealed that eight patients were diagnosed with bronchial asthma, and ten individuals had severe obstructive bronchiolitis. In 75% of asthma cases, positive challenge tests were observed following inhalation exposure to styrene which suggests a potential association between styrene exposure and the onset of respiratory conditions [20].

Another case report described a 46-year-old employee of a yacht manufacturer who presented with symptoms of shortness of breath, coughing and chest discomfort persisting for 2 months, which worsened at work. Treatment with systemic antibiotics, inhaled bronchodilators, and inhaled corticosteroids provided slight relief, but symptoms persisted. Further evaluation revealed a widespread interstitial pattern on chest X-ray and restriction loss on spirometry. Resolution of symptoms and improvement in spirometry parameters occurred only after administering oral corticosteroids and avoiding exposure to the work environment. This case suggests a potential link between the individual's symptoms and their workplace exposure to styrene which can lead to asthma and severe lung conditions in some people [21]. Styrene is a major component in the manufacture of fiberglass reinforced plastic (FRP) boats like yachts. However, vapors emitted during the application and curing of FRP boats can pose inhalation risks for nearby workers.

### Health Effects on the Nervous System

While previous studies have shown that certain MNPs can enter the brain and lead to changes in tissue structure, damage to the blood-brain barrier, and impairment of neurological function. These effects could potentially contribute to the development of neurodevelopmental issues and neurodegenerative diseases [22]. There is scarcity of case reports implicating MNPs to nervous system conditions. One of the few reported cases involved styrene, and the authors acknowledged the rarity of the finding. The case involved a 57-year-old man previously in good health who developed symptoms of peripheral neuropathy after spending two days inside a septic tank applying fiberglass resin. His symptoms were also confirmed by nerve conduction tests [23]. Fiberglass resin typically contains minuscule plastic particles that may break off during application or degrade over time. The other case involved animals experiments which reported that exposure to polystyrene microplastics leads to neurotoxic effects characterized by increased oxidative stress, damage to lipids, and the inhibition of the acetylcholinesterase enzyme [24]. Inhibition of the acetylcholinesterase enzyme has been associated with various neurological diseases and conditions. Acetylcholinesterase is responsible for breaking down the neurotransmitter acetylcholine, which is critical for communication between nerve cells in the brain and throughout the nervous system [25].

### Health Effects on the Reproductive system

Some studies have reported that certain chemicals found in MNPs, including phthalates and bisphenol A (BPA) commonly found in plastics can accumulate in the reproductive organs of mammals, causing toxic effects on the reproductive systems of men and women. In males, MNPs damage testicular and sperm structure, reduce sperm vitality, and disrupt hormonal balance. This damage results from oxidative stress, inflammation, cell death in the testes, abnormal cell processes, and hormonal axis disruption. In females, MNPs cause abnormalities in ovarian and uterine structure, disrupt hormonal balance, induce cell death in granulosa cells, disrupt the hypothalamic-pituitary-ovary axis, and lead to tissue fibrosis [26].

A study that examined 30 semen samples for microplastics using pyrolysis-gas chromatography/mass spectrometry (Py-GC/MS) and laser direct infrared spectroscopy (LD-IR) found that polyethylene (PE) and polyvinyl chloride (PVC) were the most common polymers found in semen [27]. Polystyrene microplastics (PS-MPs) have also been shown to cause significant toxic effects on the reproductive system which affects sperm quality and testosterone levels [28]. MNPs can absorb and carry phthalates which have also been reported to negatively affect egg and sperm quality [29].

In a recent study, scientists tested 4 types of sex toys: dual vibrators, external vibrators, anal toys and beads all of which interact with intimate and permeable parts of the body and reported that anal toys release the most plastic bits, followed by beads, dual vibrators, and external vibrators, the release of microplastics and nanoplastics by sex toys during use can have negative consequences for reproductive health [30]. However we found just one case report directly linking plastic use and MNPs to fertility and negative pregnancy outcomes in humans. The case involved pregnant woman in the Health Outcomes and Measures of the Environment (HOME) Study, who had a urinary bisphenol A (BPA) concentration of 583 µ g/g creatinine at 27 weeks of pregnancy. The researchers investigated potential sources of the BPA exposure and reported that daily use of plastic items and consumption of canned beverages and foods may have contributed to her elevated BPA levels since it can gain entry into the human body via various routes. Following the delivery of her infant, the initial assessments indicated no signs of abnormal neurological behavior. However, at the one-month check-up, the baby displayed some abnormalities. As a result, the child was referred to a physician for further evaluation. Fortunately, subsequent neurobehavioral tests conducted annually from ages 1 to 5 showed normal results, indicating that the child's development was not significantly affected in the long term [31].

### Health Effects on the Skeletal System

Phthalates and BPA found in plastics can also leach out from MNPs and exert toxic effects on bone cells interfering with normal bone cell function and development [32, 33]. Studies have revealed that MNPs can act as carriers of heavy metals or form complex contaminants with far reaching health consequences when absorbed by the human body [34]. A report of three cases of lead poisoning among plastics compounders at one California company were investigated. The investigation involved interviews with the affected workers, the employer, and the treating physician, as well as a review of medical records and environmental monitoring data. In addition to traditional blood lead level (BLL) measurements, noninvasive K X-ray fluorescence was employed to assess bone lead concentrations in the primary case. Upon diagnosis, the BLLs of the three affected workers were found to be 159, 114, and 108 (µ g/dL) respectively. The worker with the highest lead exposure presented with clinical symptoms including constipation, crampy abdominal pain, normocytic anemia, reversible azotemia and fatigue. Bone lead concentrations were measured in various sites, with concentrations of 102 ppm, 219 ppm, and 182 ppm in the tibia, calcaneus, and patella respectively [35]. Bone lead is an indicator of cumulative lead exposure which has been reported to be associated with adverse health consequences including neurodegenerative disease, cardiovascular disease, and higher mortality rates in aging populations [36].

### Health Effects on the Cardiovascular System

Another negative health effect of MNPs pollution manifests on the cardiovascular system. Finding from studies indicates that can adversely affect both cardiac functions and (micro)vascular sites. Direct cardiac toxicity includes abnormalities in heart rate, impairment of cardiac function, myocardial fibrosis and pericardial edema. On (micro) vascular sites, MNPs induce blood coagulation, hemolysis, thrombosis and damage to vascular endothelial cells. These effects are mediated through mechanisms such as inflammation, oxidative stress, apoptosis, pyroptosis, and interactions between MNPs and various cellular components [37]. One particular case study that on the impact of MNPs on cardiovascular morbidity and mortality involved 304 patient who were undergoing carotid endarterectomy for asymptomatic carotid artery disease,. At the end of the study it was found that those who had MNPs in their plaque were more likely to have heart attacks, strokes, or even die compared to those without MNPs [38].

### Health Effects on the Excretory System

The human excretory system is also impacted by MNPs. Studies have shown that despite being indigestible, plastic bezoars if left untreated in the digestive system can cause of gastrointestinal obstruction and potentially degrade over time due to constant motion, the acidic environment and enzymatic activity in the gut leading to the formation of MNPs that can then be absorbed into the body or excreted [39, 40].

A rare case report involved a 58-year-old female patient admitted for acute kidney injury. During the diagnostic process, a potential foreign body was identified via computerized tomography (CT) scan examination. Subsequently, the patient underwent an exploratory laparotomy procedure. During this process, plastic foreign objects were discovered within the gastrointestinal tract, notably positioned approximately 90 centimeters proximal to the ileocecal valve, along with additional foreign objects identified within the stomach [41]. Studies have shown that exposing the human kidney and liver cells to MNPs can induce toxicological problems that affects cell metabolism and cell–cell interactions which often results morphological alterations, metabolic disruptions, changes in cellular proliferation, and cellular stress responses [42].

### Further Research Needs

In view of the current findings, scarcity of studies and the widespread use of plastics in our daily routines, there is the need for empirical research preferably randomized control trials to ascertain causal relationship between MNPs and adverse health consequences. In addition, longitudinal studies involving long term follow up of people exposed to MNPs can also provide additional informal on the various adverse health effects.

## Conclusions

Health effects of MNPs exposure and treatment progress were reviewed across various systems of the body. The review captured intentional and unintentional exposures to MNPs and their components. However, case reports of the health impact of MNPs were scarce. The widespread presence of MNPs in the environment raises significant concerns regarding potential health effects on humans. Despite limited understanding, existing case reports highlight associations with adverse outcomes across various body systems. Further research is critical to comprehensively assess and mitigate the risks posed by MNPs.
